# Formulation of Self-Nanoemulsifying Drug Delivery System of Cephalexin: Physiochemical Characterization and Antibacterial Evaluation

**DOI:** 10.3390/polym14051055

**Published:** 2022-03-07

**Authors:** Ameeduzzafar Zafar, Mohd Yasir, Nabil K. Alruwaili, Syed Sarim Imam, Omar Awad Alsaidan, Sultan Alshehri, Mohammed M. Ghoneim, Ali Alquraini, Alenazy Rawaf, Mohammad Javed Ansari, Udai Vir Singh Sara

**Affiliations:** 1 Department of Pharmaceutics, College of Pharmacy, Jouf University, Sakaka 72341, Al-Jouf, Saudi Arabia; nkalruwaili@ju.edu.sa (N.K.A.); osaidan@ju.edu.sa (O.A.A.); 2 Department of Pharmacy, College of Health Science, Arsi University, Asella 396, Ethiopia; mohdyasir31@gmail.com; 3 Department of Pharmaceutics, College of Pharmacy, King Saud University, Riyadh 11451, Saudi Arabia; salshehri1@ksu.edu.sa; 4 Department of Pharmacy Practice, College of Pharmacy, Almaarefa University, Ad Diriyah 13713, Saudi Arabia; mghoneim@mcst.edu.sa; 5 Department of Pharmaceutical Chemistry, Faculty of Clinical Pharmacy, Al Baha University, Al Baha 65731, Saudi Arabia; aalquraini@bu.edu.sa; 6 Department of Medical Laboratory, College of Applied Medical Sciences-Shaqra, Shaqra University, Shaqra 11961, Saudi Arabia; ralenazy@su.edu.sa; 7 Department of Pharmaceutics, College of Pharmacy, Prince Sattam Bin Abdulaziz University, Al-Kharj 11942, Saudi Arabia; javedpharma@gmail.com; 8 Hygia Institute of Pharmaceutical Education & Research, Lucknow 226020, Uttar Pradesh, India; uvssara@gmail.com

**Keywords:** oral delivery, cephalexin, SNEDDS, anti-microbial activity, pharmacokinetic activity

## Abstract

A cephalexin (CEP) self-nanoemulsifying drug delivery system (SNEDDS) was developed in this study to improve the drug’s oral administration. The CEP-SNEDDS was made utilizing an aqueous titration method employing Lauroglycol 90, Poloxamer 188, and Transcutol-HP. Box-Behnken design (BBD) with three factors at three levels was used for optimization, and their impacts on globule size (nm), transmittance (percent), and emulsification time (s) were assessed. The optimized formulation (Opt-F3) was further tested for zeta potential, refractive index, percent transmittance, thermodynamic stability, in-vitro release, ex vivo permeability, antibacterial activity, and bioavailability. The chosen formulation (Opt-F3) had a globule size of 87.25 ± 3.16 nm, PDI of 0.25, zeta potential of −24.37 mV, self-emulsification duration of 52 ± 1.7 s, and percentage transmittance of 99.13 ± 1.5%, viscosity of 96.26 ± 2.72 cp, and refractive index of 1.29 ± 0.1. It showed a sustained release profile (94.28 ± 5.92 percent in 24 h). The Opt-F3 formulation had 3.95 times the permeability of CEP-dispersion. In comparison to CEP-dispersion, it also demonstrated greater antibacterial efficacy against tested Gram-positive and Gram-negative pathogens. The oral bioavailability of Opt-F3 is 3.48 times higher than that of CEP-dispersion, according to an in-vivo investigation. It has been determined that the prepared CEP-SNEDDS may be an advantageous carrier for CEP delivery.

## 1. Introduction

Cephalexin (CEP) is a first-generation cephalosporin derivative that is given orally. It belongs to the β-lactam class of antibiotics and is commercially available as a monohydrate. It has chemical formula C_16_H_17_N_3_O_4_S·H_2_O and a molecular weight of 365.41 g/mol [1]. It is effective in the treatment of Staphylococci and Streptococci infections [2]. It was also found to be effective against *Escherichia coli* [3]. It is listed as a key access antibiotic in the World Health Organization’s essential drug list and it is used as a second choice drug in the form of oral delivery for the effective treatment of chronic obstructive pulmonary disease (COPD), pharyngitis, skin, and soft tissue infections [4]. It is a viable alternative to penicillin for patients with penicillin hypersensitivity [5]. Cephalexin is a water-insoluble antibiotic with a half-life of 1 to 1.5 h [5]. It has a low bioavailability of 35% [6,7], and around 80% of the absorbed drug is excreted unaltered in the urine. Because of the short half-life and frequent dosage, it is necessary to maintain the therapeutic level in order to show biological activity. Furthermore, maintaining therapeutic levels in the blood is critical for short-half-life antibiotics in order to avoid resistance [8,9].

Researchers are increasingly interested in developing nanoformulations to improve the solubility and efficacy of pharmacologically active compounds. A self-nanoemulsifying drug delivery system (SNEDDS) is one of the most effective delivery system for enhancing the efficacy of poorly soluble drugs. SNEDDS is a combination of drug, oil, surfactant, and co-surfactant [10]. SNEDDS comes into contact with water, it spontaneously forms oil in water nanoemulsion (droplet size 50–250 nm) [11]. Two surfactants are used because they increase the fluidity of the interface between the dispersed droplets and the outer phase, preventing the formation of microemulsion. 

The co-surfactant increases the fluidity of the interface between dispersed droplets and the outer phase by affecting the distribution of surfactant molecules on the contact [12,13]. They are automatically self-emulsified (250 nm) when they come into contact with aqueous medium such as GI fluids and moderate agitation owing to peristalsis movement in the case of oral distribution. It increases the bioavailability of poorly soluble medicines by causing drug solubilization and absorption through the lymphatic circulation, avoiding first-pass metabolism [14]. SNEDDS has also been shown to protect drugs against enzymatic metabolism and the GIT’s sulfhydryl barrier, reducing gastric degradation resistance [15,16,17]. Therefore, many gastric labile, as well as lipophilic drugs, have been delivered through the development of SNEDDS as reported in the previous literature [15,17,18].

CEP-SNEDDS was developed for oral delivery employing oil (lauroglycol 90), surfactant (poloxamer 188), and co-surfactant (transcutol HP). The oil (lauroglycol 90) was included in the SNEDDS formulation to increase solubility and bioavailability [15]. For the preparation of SNEDDS, a combination of the utilized surfactant (Poloxamer 188) and co-surfactant (Transcutol HP) has been described [15]. To improve solubility, bioavailability, and therapeutic efficacy, several CEP formulations have been developed. The formulations like immediate and sustained release formulations [3], controlled release matrix tablets [8], silica micro-particles [19], non-ionic microemulsions [5], extended-release matrix tablets [7,20], and gastro-floating tablets [6,21] have been developed and tested. The objective of the current research was to develop a CEP SNEDDS and evaluate it by a titration method. A preliminary screening study was conducted to select the best oil, surfactant, and co-surfactant. After the selection of the formulation components, it was further optimized by a 3^3^ Box-Behnken design (BBD). The developed formulations were characterized using globule size, emulsification time and percentage transmittance, etc. A comparative in-vitro release study was conducted and finally, antimicrobial and pharmacokinetic studies were also conducted.

## 2. Materials and Methods

### 2.1. Materials

Cephalexin was procured from Spectrum Pharma Ltd. (Roorkee, UK). Labrafac WL, Labrasol, Caprol PGE-860, Capmul MCM, Labrafil M1944 CS and Transcutol HP were purchased from Gattefosse (Mumbai, India). The different oils (eucalyptus oil, almond oil, sunflower oil, ethyl oleate) were purchased from SD-Fine Chemicals (Mumbai, India). BASF India Ltd. (Bandra East, Mumbai, India) provided Lauroglycol 90, Cremophor RH 40, Solutol HS15, and Span 20. Other chemicals like polyethylene glycol-400 (PEG-400), PEG200 and HCl were acquired from Acros Organics (Mumbai, India). Poloxamer188 and other chemicals were obtained from Sigma Aldrich (St-Louis, MO, USA).

### 2.2. Methods

#### 2.2.1. Preliminary Screening

The equilibrium solubility method was used to select the oil, surfactant, and co-surfactant [22]. The solubility of CEP in each components was evaluated in order to select the oil, surfactant, and co-surfactant. The screening was performed in variety of oils, surfactants, and co-surfactants. In a clean glass vial, 1.5 mL of each oil, surfactant, and co-surfactant was added, along with an excess of CEP. The vials were capped properly and shaken on a mechanical shaker at 37 ± 0.5 °C for 72 h. The samples were centrifuged at 10,000 rpm for 5 min and then supernatant was collected. The amount of CEP dissolved in each component was analyzed at 262 nm with the help of UV spectrophotometer (Shimadzu 1800, Kyoto, Japan) after a suitable dilution. 

#### 2.2.2. Pseudo Ternary Diagram

The purpose of making a pseudo ternary diagram was to choose emulsification efficiency and a reasonable oil, surfactant, and co-surfactant concentration [23]. This was achieved using the aqueous titration method. To prepare variable ratios, a combination of surfactant and co-surfactant was used (1:0, 1:1, 1:2, 2:1, 1:3). Smix was mixed with oil in various ratios ranging from 9:1 to 1:9, and then titrated with a predetermined amount of double-distilled water. The presence of turbidity was carefully inspected visually after the complete reaction. Using a phase diagram, the self-nanoemulsifying zone was calculated (triangular co-ordinate software). The ratio of oil, surfactant, and co-surfactant was calculated using the maximum area. The three levels of each component were selected for further investigation (Table 1). 

#### 2.2.3. Optimization

Box Behnken design (BBD, DoE Software, Stat-Ease, Minneapolis, MN, USA) was used to analyze the effect of independent constraints (oil, surfactant and co-surfactant) on the different dependent responses (globule size, % transmittance, emulsification time). It works on the principle of response surface methodology and gives fewer compositions than a parallel response surface design (central composite design). It revealed the effect of formulation constraints individually, their interactions, and quadratic expressions [24]. The three factors at three levels, depicted a total of seventeen runs with five similar compositions to assess the error. The data was applied to various models, viz linear, 2F1, and quadratic to explore the influence of independent variables. ANOVA, polynomial equations, and surface plots were generated and explored the effect of independent variables on the dependent variables. 

#### 2.2.4. Preparation of CEP-Loaded SNEDDS

The aqueous titration method was used for preparation of CEP-SNEDDS [22]. A total of seventeen formulations were developed as per the composition given in Table 2. Oil (A), surfactant (B), and co-surfactant (C) were chosen and mixed in a glass vial to form a homogenous mixture (F1–F17). The samples (F1–F17) were vortexed with CEP (100 mg) to obtain a uniform homogenous mixture. The formulations were stored at room temperature for further characterization [25]. 

#### 2.2.5. Evaluation of CEP SNEDDS

##### Droplet Characterization

The particle size analyzer (ZEN-3600, Malvern, Malvern Instruments, Westborough, MA 01581, USA) was used to evaluate droplet size, polydispersibility index and zeta potential. CEP-SNEDDS were diluted (100-fold) with deionized water and transferred to a cuvette for evaluation. The surface morphology was evaluated by transmission electron microscopy (TEM, Mic JEM1011, JEOL, Tokyo, Japan). A drop of CEP-SNEDDs was placed on a carbon-coated copper grid and kept for a few minutes. Then phosphotungistic acid as staining agent was added to the sample. Any excess was removed with filter paper and the sample was air-dried. The grid was placed in the TEM instrument and the image was captured.

##### Detection of Self-Nanoemulsification Time

Self-emulsification time is the time required for the sample to become self-emulsified with distilled water. The self-emulsification process was visually evaluated for the emulsification property [22]. The study was performed with a USP paddle-type apparatus (Sotex AG, Aesch, Switzerland) to determine the emulsification time. Distilled water (500 mL) was placed in the vessel, and the temperature and rotation speed were set at 37 ± 0.5 °C and 50 rpm, respectively. CEP-SNEDDS was added to the media and then the emulsification time was noted.

##### Percentage Transmittance

CEP-SNEDDS was diluted 100-fold with distilled water and the percentage transmittance was determined by UV visible spectrophotometer at 638 nm. The study was performed in triplicate for each formulation.

#### 2.2.6. Evaluation of Optimized Formulation

##### Thermodynamic Stability

The stability of CEP-SNEDD (Opt-F3) was evaluated by applying various stressors, i.e., centrifugation, heating cooling and freeze-thawing.

Centrifugation: Opt-F3 was diluted (1:100) with water and centrifuged (EMA 200, Hettich, Tuttlingen, Germany) at 7000 rpm for 20 min. Then, it was observed visually for any phase separation, phase inversion and creaming [26]. 

Heating and cooling study: This study was performed by taking Opt-F3 and kept at 4–40 °C for 48 h. Then the samples were assessed for instability (phase separation, creaming and phase inversion). 

Freeze-thaw stability: Reconstituted optimized SNEDDS (Opt-F3) was kept under temperature range of −20 to 25 °C for 48 h and examined visually for any instability.

##### Viscosity and Refractive Index

The viscosity of optimized SNEDDS (Opt-F3) was determined using a Brookfield viscometer (DVT2 viscometer, Brookfield Middleboro MA) at 40 rpm using a 31S spindle size. Refractive index was measured by a refractometer (Kruss, Abbes-refractometer, Service-Hotline, Germany) at 25 ± 2 °C.

##### DSC Analysis

DSC analysis was performed for CEP, Lauroglycol 90, Poloxamer 188, Transcutol HP, and optimized SNEDDS (Opt-F3) using a DSC instrument (Mettler Toledo, Toledo, OH, USA). In brief, about 5 mg of each sample was packed into an aluminum pan and placed into the DSC instrument. The samples were examined between 20–450 °C at a heating rate of 10 °C/min with a continuous supply of nitrogen at 50 mL/min. An empty aluminum pan was used as a reference. The thermogram was collected to interpret the changes in sample behaviour.

#### 2.2.7. Drug Release Study

The study of CEP from Opt-F3 was studied in a USP paddle dissolution apparatus. The release medium (0.1 *N* HCl, pH-1.2, 900 mL) was placed into a basket and maintained at 37 ± 0.5 °C to simulate stomach condition. The sample was placed in a dialysis bag (MW CO- 12 kDa) and tied from both ends. The dialysis bag was attached to a paddle, dipped into a dissolution medium and rotated at 100 rpm. At a predetermined time, 5 mL of the sample was withdrawn, suitably diluted and filtered. The absorbance was analyzed by UV visible spectrophotometer at 262 nm [6,27]. The release was calculated and a graph between % drug release and time was plotted. A similar study was performed for pure CEP in similar condition for comparison. The release data fitted to various kinetic models to evaluate the release kinetics and release mechanism [24,28].

#### 2.2.8. Ex-Vivo Permeation Study

The study was performed using goat intestine collected from a slaughterhouse. The intestine was cleaned in a biological Ringer solution and filled with the formulation. The intestine was tied at both ends and dipped in biological Ringer solution (100 mL). The study was conducted on a magnetic stirrer (50 rpm) and the temperature was maintained at 37 ± 0.5 °C. At a predetermined time, 2 mL sample was taken up to 6 h and drug permeation was analyzed [6,29], using UV visible spectrophotometry at 262 nm. The same procedure was repeated with pure CEP-dispersion in water. A graph of the amount of drug permeated versus time was plotted. The different parameters i.e., flux (J, μg/cm^2^/h), permeability coefficient (PC) and enhancement ratio (ER) were calculated using the following equations [27,30]:

(1) $$\mathrm{Flux}=\frac{\mathrm{Slope}}{\mathrm{Diffusional}\;\mathrm{area}}$$

(2) $$\mathrm{PC}=\frac{\mathrm{Flux}}{\mathrm{Initial}\;\mathrm{conc}.}$$

(3) $$\mathrm{ER}=\frac{\mathrm{Flux}\;\mathrm{of}\;\mathrm{CEP}\;\mathrm{SNEDDS}}{\mathrm{Flux}\;\mathrm{of}\;\mathrm{CEP}\;\mathrm{dispersion}}$$

#### 2.2.9. Antimicrobial Study

The antimicrobial study was conducted on *S*. *aureus* (Gram positive,) and *E. coli* (Gram negative). The study was performed by the diffusion method [31]. The efficacy was determined in the terms of zone of inhibition (ZOI) and findings were compared between opt-F3 and pure CEP-dispersion. The test organisms were grown on sterile Luria Bertani broth and the plates were cultured with 100 µL of bacteria. Ten mL of nutrient agar were transferred to the plates and allowed to solidify. Three wells were made on each plate and sample (100 µL) was placed to each well. The plates were incubated for 48–72 h in an incubator set at 37 ± 0.5 °C [19,32]. Each experiment was conducted in triplicate and the zone of inhibition was then measured. 

#### 2.2.10. Bioavailability Study

The study was performed in albino Wistar rats (200–250 gm of either sex). The animal study protocol was approved by the Institutional Animal Ethical Committee of the Hygia Institute of Pharmaceutical Education and Research (Lucknow, India; IAEC No. HIPER/IAEC/69/21/04). Rats were obtained from the animal house and kept under the recommended conditions with free access to food and water. Before the experiment, the animals were allowed to acclimatize to normal conditions (25 ± 2 °C, and 12 h day/night cycle) for a week. The rats were divided into two groups, where Group I received pure CEP dispersion and Group II received the optimized SNEDDS (Opt-F3) (equivalent to 22 mg/kg of CEP) orally. The dose was calculated based on the body surface area in comparison to humans. After oral administration, blood samples were withdrawn from the retro-orbital plexus into a pre-heparinized tube at different time points (0, 0.5, 1, 2, 4, 6, and 12 h). The blood was centrifuged at 4000 rpm for 15 min (Remi Centrifuge, Mumbai, India) and the plasma separated.

#### 2.2.11. Extraction of CEP from Plasma

In a tube, the plasma sample (0.2 mL) was combined with perchloric acid for deproteination (0.2 mL, 70%) and vortexing (2 min). Finally, the sample was centrifuged for 10 min at 12,000 rpm, and the supernatant recovered. The extract was dissolved in methanol after the supernatant was dried under vacuum. HPLC (LC 10 AD model, Shimadzu, Tokyo, Japan) was used for the analysis. The instrument was connected to a gradient flow pump that included an auto sampler and C_18_ column (D150 × 4.6 mm, 5 µm). The sample (20 µL) was injected into the column and 0.01 mol/L sodium acetate buffer/methanol (25:75, *v/v*) was used as mobile phase with a flow rate of 1 mL/min. detection was carried out with a UV-detector set at 262 nm.

#### 2.2.12. Pharmacokinetic Study

For the above said method, plasma drug concentration at various times was obtained. A plot of plasma concentration vs. time was constructed and different pharmacokinetic parameters Cmax, Tmax, AUC, half-life (t_1/2_), elimination rate constant (Kel) etc. were calculated. Finally, the relative bioavailability was determined in comparison to CEP dispersion:

(4) $$\mathrm{Relative}\;\mathrm{Bioavailability}\;\left(\%\right)=\frac{\left({\mathrm{AUC}}_{0-\infty }\right)\;\mathrm{of}\;\mathrm{SNEDDS}}{\left({\mathrm{AUC}}_{0-\infty }\right)\;\mathrm{of}\;\mathrm{drug}\;\mathrm{dispesion}}\times 100$$

#### 2.2.13. Statistical Analysis

All results were expressed in the terms of mean ± SD. GraphPad Prism (GraphPad Software, San Diego, CA 92108, USA) was used for statistical analysis. For the comparison analysis, Student’s-test and analysis of variance (ANOVA) with Tukey Karman post-test was applied. 

## 3. Results and Discussion

### 3.1. Preliminary Screening

**Figure 1:**
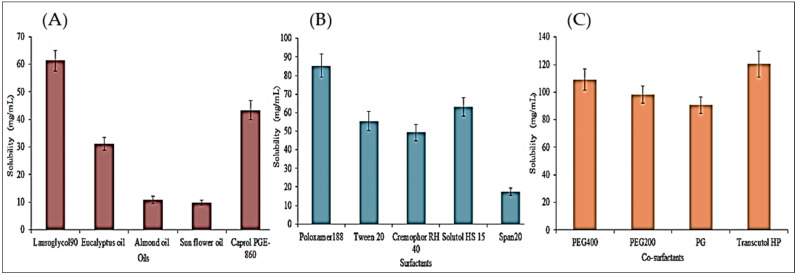
Solubility data of CEP in different oils (**A**), surfactants (**B**) and co-surfactants (**C**). Study performed in triplicate and results shown as mean ± SD.

Figure 1A–C shows the solubility of CEP in different oils, surfactants, and co-surfactants, and selection was made based on the highest solubility. Lauroglycol l90 > Caprol PGE-860 > eucalyptus oil > almond oil > sunflower oil is the order of CEP solubility in different oils (Figure 1A). Poloxamer188 > Solutol HS 15 > Tween 20 > Cremophor RH 40 > Span 20 and Transcutol HP > PEG400 > PEG200 > PG is the order of CEP solubility in surfactant and co-surfactant (Figure 1B,C). Lauroglycol 90 (61.37 ± 3.72 mg/mL), Poloxamer 188 (85.26 ± 6.28 mg/mL), and Transcutol HP (120.32 ± 9.36 mg/mL) as oil, surfactant, and co-surfactant, respectively. Poloxamer 188 is a surfactant that is hydrophilic and has an HLB value of 29. It allows SNEDDS to quickly emulsify when they come into contact with water.

### 3.2. Preparation of the Pseudo Ternary Diagram

**Figure 2:**
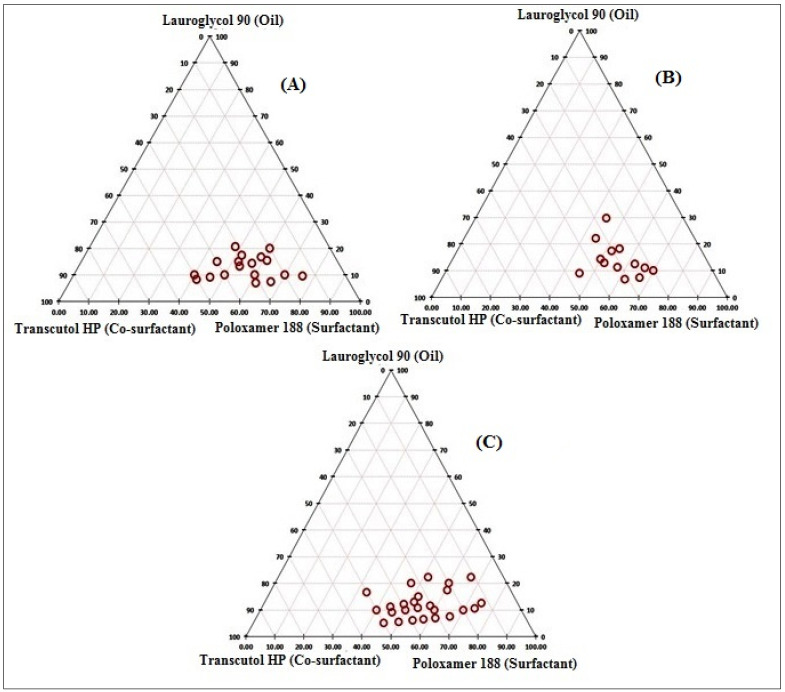
Pseudo ternary phase diagram of different Smix ratios: (**A**). 1:1; (**B**) 1:2; (**C**). 2:1.

The goal of constructing the phase solubility diagram was to get a large enough self-emulsification zone [15,33]. To obtain a clear and stable SNEDDS, multiple surfactant and co-surfactant ratios (1:1, 1:2, 2:1) were examined, as well as varied oil ratios (9:1, 1:9), as shown in Figure 2. The best surfactant and co-surfactant ratio (Smix) was discovered to be 2:1, which demonstrated a clear zone when compared to the other surfactant and co-surfactant ratios of 1:1 and 1:2. The absorption of surfactant at the oil-water interface, which is responsible for the lowering of interfacial tension, could explain the transparency of the zone in the Smix 2:1 ratio [15,34], so from the phase diagram, oil (Lauroglycol 90, 5–28%, *w/w*), surfactant (Poloxamer 188, 32–78%, *w/w*) and co-surfactant (Transcutol HP, 8–52%, *w/w*) was used for the optimization and preparation of SNEDDS.

### 3.3. Optimization

To find the best composition, three independent variables were used: oil (Lauroglycol 90, 5–28%), surfactant (Poloxamer 188, 32–78%), and co-surfactant (Transcutol HP, 8–52%. This data was fed into the optimization model, yielding 17 different compositions, including five common formulas (center point). The data was fitted to various models, and the quadratic model proved to be best fit as seen in Table 3.

### 3.4. Effect of Variables on Globule Size (Y_1_, nm)

As presented in Table 2, globule size (Y_1_) of CEP- SNEDDs (F1–F17) was found to be in the range of 30.66–205.84 nm. The data was added to the design and the polynomial Equation (5) was generated, as shown below:

Globule size (Y_1_, nm) = +108.99 + 57.79A − 28.91B − 222.44C − 11.73AB + 0.81AC − 5.448BC − 4.03A^2^ + 5.24B^2^ + 5.04C^2^ (5)

**Figure 3:**
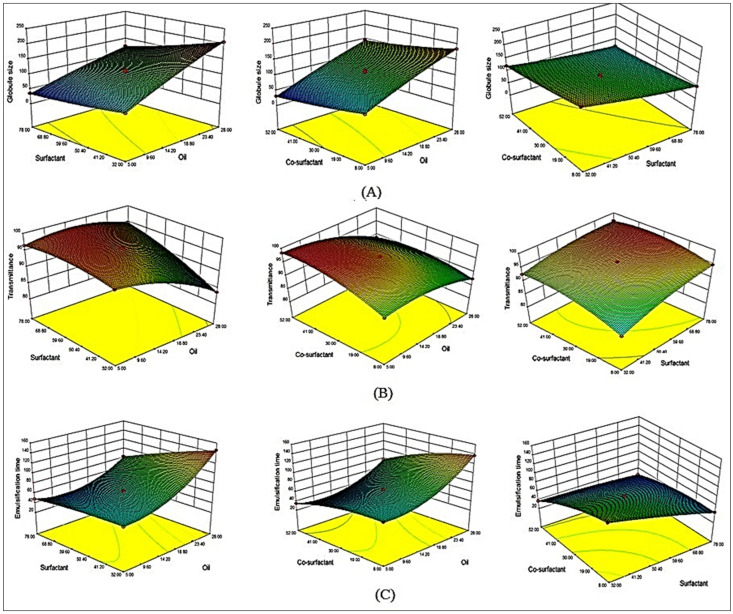
(**A**) 3D surface response diagram of CEP-SNEDDS indicating the influence of independent variables on (**A**). Globule size as Y_1_, (**B**). Percent transmittance as Y_2_, (**C**). Emulsification time as Y_3_.

It may be inferred from the above equation that model term A had a positive effect on the size, but model terms B and C had a negative effect. The model terms A, B, C, AB, BC, A^2^, B^2^, and C^2^ were all found to be significant (*p* < 0.05), but AC was found to be non-significant (*p* > 0.05). The “Pred R-Squared (0.9885)” and “Adj R-Squared (0.9957)” were found to be in reasonable agreement. The quadratic model, as indicated in Table 3, was the best-fitting model. The first variable oil (A) had a positive effect on globule size, while the second variable surfactant (B) and the third variable co-surfactant (C) had a negative effect. The size of globules increases as oil content rises. Due to partial emulsification in the presence of insufficient surfactant and co-surfactant concentrations, the size of the globules increased (F1–F2) as the oil concentration increased (F1–F2). On other hand, as concentration of surfactant and co-surfactant increases, the size of the globules decreased (F1–F3 and F6–F8). This could be due to surfactant and co-surfactant accessibility at the oil/water interface, which helped to reduce interfacial tension and contribute to system stability [16,18]. Similar findings have already been reported in literature [22,23]. The surfactant and co-surfactant together showed opposite effect, with globule size decreasing as the concentration increased. This could be due to the significant effect of either surfactant or co-surfactant on globule size, as both had the same effect. When both oil and surfactant are increased, globule size falls due to surfactant’s dominant influence over the oil. However, increasing the oil and co-surfactant concentrations together increases the viscosity of the medium due to the dominant influence of oil over the co-surfactant. The 3D graphics show the effect of all independent factors on globule size, including oil, surfactant, and co-surfactant (Figure 3A).

### 3.5. Effect of Variables on Transmittance (Y_2_, %)

The transmittance (%, Y_2_) for all developed CEP- SNEDDS was found to be in the range of 83.73 (F2)–99.39% (F7) as shown in Table 2. A polynomial equation (Equation (6)) was used to interpret effect of independent variables on the transmittance (%).

Transmittance (Y_2_, %) = +96.29 − 4.79 A + 3.88B + 2.50C + 2.95 AB − 1.28 AC + 0.81BC − 2.39 A^2^ − 1.24B^2^ − 2.19 C^2^ (6)

It may be inferred from the above equation that the term A has a negative impact, and the terms B as well as C, had a favorable effect on the transmittance (%, Y_2_). The transmittance (%) decreased as the oil content increased (F1–F2). This could be caused by an insufficient amount of surfactant and co-surfactant [29,33]. On the other hand, when the concentration of surfactant and co-surfactant increases, the transmittance also increases (F1–F3 and F6–F8). This could be due to surfactant and co-surfactant accessibility at the oil/water interface, which helped to reduce interfacial tension and increase transmittance. A similar finding was reported for an atorvastatin calcium SNEDDS [23]. The combined impact also revealed that when the concentration of oil (A) and surfactant (B) rises, the transmittance is also increased. As a result, as oil and co-surfactant concentrations increase, the transmittance (percent) rises. This could be attributed to liquid crystal formation as well as an increase in the system’s viscosity. 3D images shows effect of all independent factors on transmittance, including oil (A), surfactant (B), and co-surfactant (C) (Figure 3B). All terms A, B, C, AB, AC, BC, A^2^, B^2^, and C^2^ were considered to be important in this case. The “Pred R-Squared (0.9988)” and “Adj R-Squared (0.9995)” were found to be in reasonable agreement. Table 3 shows the statistical summary of the best-fitted model, which was found to be a quadratic one.

### 3.6. Effect of Variables on Emulsification Time (Y_3_, s)

The emulsification time of prepared CEP- SNEDDs was found to be in range 25 s (F12)–146 s (F2) (Table 2). The data fed were the actual data of emulsification time (Y_3_, s) and the following Equation (7) was generated:

Emulsification time (Y_3_, s) = +64.20 + 28.38*A − 19.50*B − 20.38*C − 11.19*A*B − 8.75*A*C + 12.50*B*C + 21.72*A^2^ − 2.78*B^2^ − 8.91*C^2^ (7)

In the preceding polynomial equation, the independent variable, oil (A), had a positive effect on emulsification time, whereas surfactant (B) and co-surfactant (C) had a negative effect (Y_3_, s). Oil (A) has a positive effect on emulsification time whereas surfactant (B) and co-surfactant (C) have a negative effect (Y_3_, s). The emulsification time increased when the oil concentration (A) was raised (F1–F2). This could be due to a lower concentration of surfactant and co-surfactant. However, as concentration of surfactant (B) and co-surfactant (C) increased, the emulsification time (Y_3_) decreased (F1–F3 and F6–F8). This could be due to surfactant and co-surfactant accessibility at oil/water interface, which reduces interfacial tension and hence reduces emulsification time. Similar findings have already been reported in the literature [23,35]. Due to the dominant effect of surfactant, the emulsification time reduced as the oil (A) and surfactant (B) were increased. The 3D images illustrate the effect of all independent factors on transmittance, including oil, surfactant, and co-surfactant (Figure 3C). The significant model terms in this situation were A, B, C, AB, AC, BC, A^2^, B^2^, C^2^. The “Pred R-Squared (0.9968)” and “Adj R-Squared (0.9992)” were found to be in reasonable agreement. Table 3 tabulates analysis data for quadratic model, which was shown to be best-fitted model.

### 3.7. Point Prediction for Optimization

For the point prediction, we used Formula F13 from Table 2. Further adjustments in composition were made in a point prediction, and their effects on globule size (nm), transmittance (percent), and emulsification time were recorded (s). Table 4 shows the alterations that were made in the composition with their findings. With oil at 14%, surfactant at 59%, and co-surfactant at 32%, the formulation opt-F3 was chosen as the best. Predicted value of globule size, transmittance, and emulsification time were found to be 84.39 nm, 98.84%, and 49.44 s, respectively. The experimental values of globule size, transmittance, and emulsification time was found to be 87.25 ± 3.16 nm, 99.13 ± 0.5 percent, and 52 ± 1.7 s, respectively. The results revealed that the percent variation between the actual and experimental values was quite low, indicating that model was well fitted.

### 3.8. Evaluation of CEP SNEDDS

#### 3.8.1. Droplet Characterization

**Figure 4:**
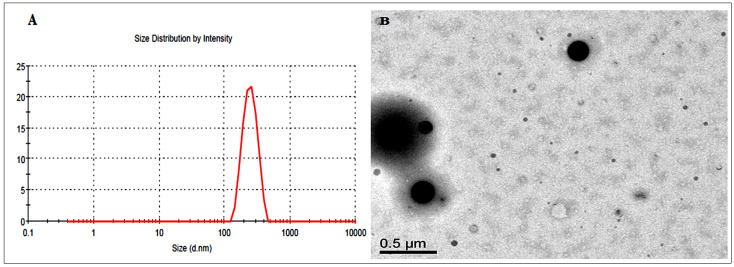
Globule size image (**A**). TEM image (**B**) of optimized CEP-SNEDDS (Opt-F3).

The globule size, PDI and zeta potential for the optimized formulation (Opt-F3) were found to be 87.25 ± 3.16 (Figure 4A), 0.25, and −24.37 mV, respectively. The low PDI value (<0.5) indicates desired stability of Opt-F3. TEM images showed spherical shaped globules with a uniform surface (Figure 4B).

#### 3.8.2. Characterization of Optimized SNEDDS (Opt-F3)

The CEP-SNEDDS-opt was subjected to a variety of stressful conditions, including centrifugation, heating, cooling, and a freeze-thaw cycle, among others (Opt-F3). Even after the induction of stress conditions, the formulation was found to be physically stable following emulsification, with no indications of instability such as creaming, cracking, or phase separation. At 25 ± 2 °C, the improved formulation (Opt-F3) had a viscosity of 96.26 ± 2.72 cps and a refractive index of 1.29 ± 0.17.

#### 3.8.3. DSC Analysis

**Figure 5:**
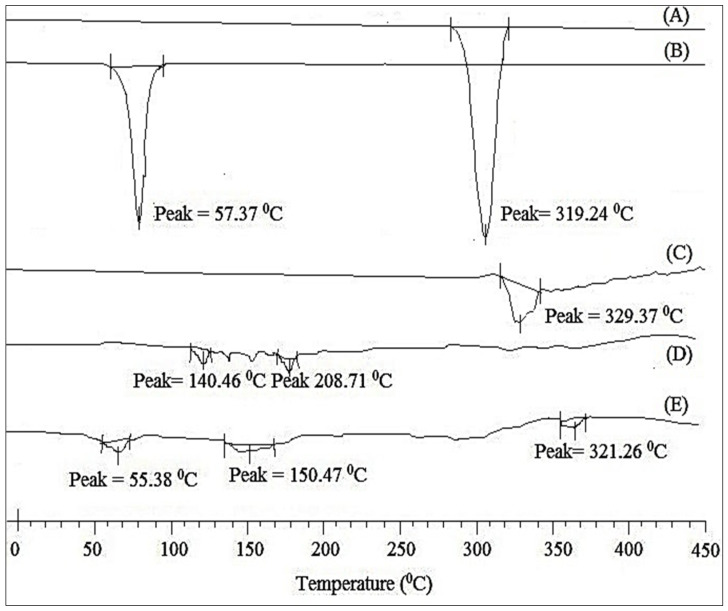
DSC thermogram of (**A**). CEP; (**B**). Poloxamer 188; (**C**) Lauroglycol 90; (**D**) Transcutol-HP; (**E**) CEP-SNEDDS (Opt-F3).

The thermograms in Figure 5A–E show the results of our DSC investigation of pure CEP, CEP-SNEDDS-opt, and the utilized components. The crystalline character of CEP was demonstrated by a strong peak at 319.24 °C (Figure 5A). The thermodynamic peak for Poloxamer was observed at 57.37 °C, whereas a wide peak for Lauroglycol 90 was seen at 329.57 °C (Figure 5B,C). At 140 °C and 210 °C, Transcutol HP showed minor peaks (Figure 5D). The CEP peak was not visible in the Opt-F3 formulation (Figure 5E). Opt- F3’s thermogram, on the other hand, showed fewer strong peaks. The complete solubilization of the drug in SNEDDS components was revealed by the lack of a prominent endothermic peak of CEP in SNEDDS [36,37].

#### 3.8.4. In-Vitro Drug Release

**Figure 6:**
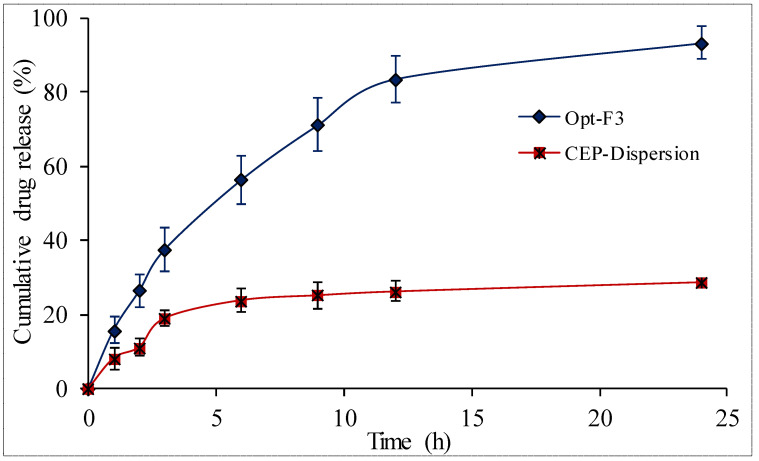
In-vitro drug release data of CEP-Dispersion and optimized CEP-SNEDDS (Opt-F3). The study performed in triplicate and data shown as mean ± SD.

The release study of Opt-F3 and pure CEP dispersion was carried out, and the results are plotted in Figure 6. Opt-F3 demonstrated a rapid release of 21.47 ± 3.83% CEP in the first 2 h, followed by a protracted release of 94.28 ± 5.92% in 24 h. The presence of CEP on the surface of SNEDDS could explain the initial rapid release. The entrapped drug within SNEDDS, on the other hand, allowed for longer-lasting drug release. In the same period, the drug release from CEP dispersion was determined to be very low at 26.76 ± 3.28%. It could be due to CEP’s low solubility. The release data of the Opt-F3 formulation was submitted to various kinetic models, with a first-order one proving the best fit (R^2^ = 0.9815). A non-Fickian transport kind of release mechanism was demonstrated by the release exponent of *n* = 0.7128.

#### 3.8.5. Ex-Vivo Permeation Study

The amount of CEP released from Opt-F3 was considerably (*p* < 0.01) larger than that of CEP-dispersion, according to the permeation study results. The Opt-F3 formulation flux (J) was determined to be 11.24 ± 2.41 µg/cm^2^/h, which is larger than CEP-dispersion flux (2.84 ± 0.35 µg/cm^2^/h). For the Opt-F3 formulation and CEP-dispersion, the permeability coefficients (PC) were found to be 0.022 cm/s and 0.0057 cm/s, respectively. The ER ratio was also calculated, and the results revealed that Opt-F3 had a flux that was 3.95 times higher than that of CEP-dispersion. These results were made possible by the presence of surfactants and co-surfactants, which are responsible for solubility of poorly soluble drugs as well as the opening of the intestinal membrane’s tight junction.

### 3.9. Antimicrobial Study

**Figure 7:**
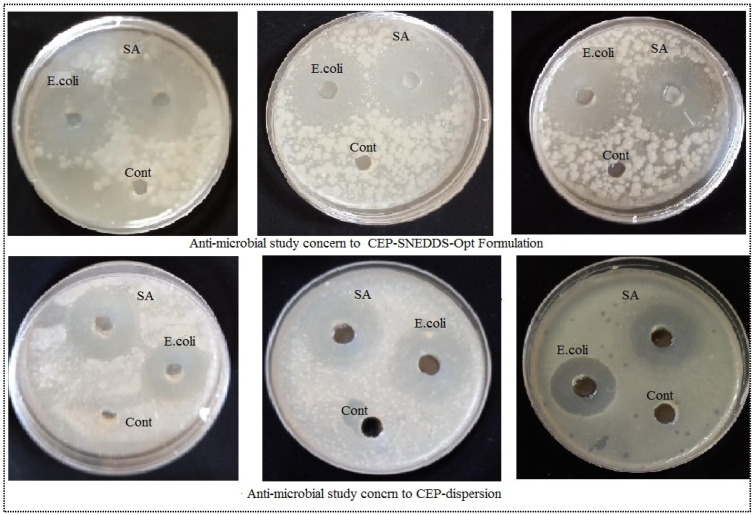
Antimicrobial activity image of CEP-SNEDDS-opt and CEP-dispersion formulation against *S. aureus* and *E. coli*.

To test the effectiveness of Opt-F3 and CEP-dispersion, antibacterial activity was performed against *S. aureus* and *E. coli* bacteria and results shown in Figure 7. The zone of inhibition (ZOI) of the Opt-F3 formulation was 15.27 ± 1.52 mm against *S. aureus* and 10.83 ± 1.37 mm against *E. coli*. Against *E. coli* and *S. aureus* administration of CEP-dispersion resulted in a significant (P0.05) change in ZOI, with a ZOI of 7.39 ± 1.23 against *S. aureus* and 5.13 ± 0.98 against *E. coli* being found. According to the findings, the produced SNEDDS had enhanced activity due to the better solubility of CEP in the presence of oil, surfactant, and co-surfactant. Because of the nano-droplet size and increased solubility of CEP, the SNEDDS have greater activity. The larger effective surface area available for drug absorption is due to the nanosize range. It also resulted in increased CEP solubility and a higher amount of medication on the target side to trigger the effect. CEP works by breaking down the microorganisms’ cell walls. Internalization of the medication in the cell wall of the microorganism occurs, resulting in cell lysis via blocking the interaction between N-acetylmuramic acid and N-acetylglucosamine [38,39].

### 3.10. Bioavailability Study

A pharmacokinetic investigation was carried out on albino Wistar rats following a single oral dose of SNEDDS-opt (Opt-F3) and CEP-dispersion, with the results presented in Table 5. The Cmax of the formulation Opt-F3 was 7.68 ± 0.72 g/mL, which was significantly greater (*p* < 0.05) than that of CEP-dispersion (4.68 ± 0.57 g/mL). Tmax was likewise found to be higher (4 h) than that of CEP-dispersion (2 h), indicating that CEP was released continuously from SNEDDS. Because of the sustained CEP release from SNEDDS, the half-life (t_1/2_) was observed to be substantially (*p* < 0.05, 5.9 h) longer than with CEP-dispersion (1.4 h). The AUC_0–t_ and AUC_0–∞_ values were determined to be 71.37 ± 4.28 g·h/mL and 77.31 ± 4.87 g·h/mL, respectively, which were considerably (*p* < 0.05) higher than the CEP-dispersion values of 20.5 ± 3.76 g·h/mL and 20.73 ± 3.76 g·h/mL. Opt-F3 has an elimination rate constant (Kel) of 0.12 h^−1^, which is significantly (*p* < 0.05) lower than that of CEP-dispersion. Opt-F3 had a 3.48-fold better percent bioavailability than CEP-dispersion. This suggested that the SNEDDS formulation could be a viable option for improving CEP oral bioavailability.

## 4. Conclusions

CEP-loaded SNEDDS for oral delivery of the drug was developed successfully. The formulation exhibited acceptable globule size, high transmittance, and short self-emulsification time. The in-vitro drug release for the optimized formulation (Opt-F3) was significantly higher than that of pure drug (CEP) dispersion. Moreover, the optimized formulation exhibited three times better permeation than that of CEP dispersion. The formulation Opt-F3 exhibited a greater antimicrobial potential in comparison to pure CEP-dispersion. The *in-vivo* study indicated that the oral bioavailability of developed formulation was 3.48-fold higher than that of CEP-dispersion. SNEDDS displayed the desired stability under the stated conditions studied. Finally, it could be concluded that the developed CEP-SNEDDS was proved to be a promising carrier for CEP delivery in the treatment of microbial disorders.

## Figures and Tables

**Table 1:** Formulation variables and their responses used to optimize CEP SNEDDS.

Variables	Levels		
	Low (−)	Medium (0)	High (+)
A = Oil (%, *w/w*)	5	16.5	28
B = Surfactant (%, *w/w*)	32	55	78
C = Co-surfactant (%, *w/w*)	8	30	52
Responses	Goals		
Y_1_ = Globule size (nm)			
Y_2_ = Transmittance (%)			
Y_3_ = Emulsification time (s)			

Oil (Lauroglycol 90); Surfactant (Poloxamer 188); Co-surfactant (Transcutol HP).

**Table 2:** Composition of different CEP-SNEDDs with their results.

S.No	Oil Conc. (%)	Surfactant Conc. (%)	Co-Surfactant Conc. (%)	Globule Size (nm)	Transmittance (%)	Emulsification Time (s)
F1	5.0	32	30	67.53	94.78	61
F2	28.0	32	30	205.84	83.73	146
F3	5.0	78	30	36.02	96.75	48
F4	28.0	78	30	123.41	94.65	83
F5	5.0	55	8	71.04	91.8	64
F6	28.0	55	8	184.28	87.6	137
F7	5.0	55	52	30.66	99.39	35
F8	28.0	55	52	146.04	89.77	77
F 9	16.5	32	8	176.26	87.04	108
F10	16.5	78	8	100.73	94.73	44
F11	16.5	32	52	120.93	92.62	37
F12	16.5	78	52	71.15	97.06	25
F13 *	16.5	55	30	114.71	96.06	64
F14 *	16.5	55	30	107.04	96.48	63
F15 *	16.5	55	30	109.74	96.26	65
F16 *	16.5	55	30	106.26	96.26	65
F17 *	16.5	55	30	107.21	96.39	64

* Common composition.

**Table 3:** Statistical summary for the best fit model shown by different selected responses.

Terms	Globule Size (Y_1_, nm)	Transmittance (Y_2_, %)	Emulsification Time (Y_3_, s)
R^2^	0.9981	0.9995	0.9997
Adjusted R^2^	0.9957	0.9988	0.9992
Prediced R^2^	0.9885	0.9966	0.9968
Model F-Value	415.79	1475.42	2276.38
Model *p*-value	<0.0001	<0.0001	<0.0001
Lack of fit F-value	0.62	0.67	1.55
Lack of fit *p*-value	0.6382 *	0.6121 *	0.3329 *
Adequate Precision	73.204	130.229	168.648

* Non-significant.

**Table 4:** Point prediction optimized composition CEP-SNEDDS.

S.No	Composition O: Sur: Co-Sur	Actual value			Predicted Value		
		Y_1_ (nm)	Y_2_ (%)	Y_3_ (s)	Y_1_ (nm)	Y_2_ (%)	Y_3_ (s)
Opt-F1	16.5:55.0:30	114.71 ± 3.27	96.06 ± 1.25	64 ± 2	108.99	96.29	64.20
Opt-F2	14.0:58.0:30	97.27 ± 2.35	96.38 ± 1.93	57.26 ± 2	91.33	97.28	56.19
Opt-F3	14.0:59.0:32	87.25 ± 3.16	99.13 ± 0.5	51.82 ± 1	84.39	98.84	49.44

O = Oil, Sur = Surfactant, Co-sur = Co-surfactant.

**Table 5:** Pharmacokinetic parameters of CEP-SNEDDS (Opt-F3) and CEP dispersion after single-dose oral administration. The study performed with six rats in each group and data given as mean ± SD.

Pharmacokinetic Parameters	CEP-SNEDDS-opt (Opt-F3)	CEP Dispersion
C_max_ (ng/mL)	7.69 ± 0.72	4.69 ± 0.57
T_max_ (h)	4	2
AUC_0–24_ (µ·h/mL)	71.37 ± 4.28	20.50 ± 3.76
AUC_0–∞_ (µ·h/mL)	77.31 ± 4.87	20.73 ± 3.76
AUMC_0–24_ (µg·h^2^/mL)	534.50	86.90
AUMC_0–∞_ (µg·h^2^/mL)	727.70	93.33
Half life (t_1/2_ h)	5.91 ± 0.15	1.24 ± 0.12
Elimination rate constant (h^−1^)	0.12± 0.05	0.21 ± 0.03
MRT (h)	9.41 ± 0.45	4.50 ± 0.5

## Data Availability

The data presented in this study are available on request from the corresponding author.
